# Motivations for people with cognitive impairment to complete an advance research directive – a qualitative interview study

**DOI:** 10.1186/s12888-020-02741-7

**Published:** 2020-07-08

**Authors:** Karin Jongsma, Julia Perry, Silke Schicktanz, Katrin Radenbach

**Affiliations:** 1 Department of Medical Ethics and History of Medicine, University Medical Center Göttingen, Humboldtallee 36, 37073 Göttingen, Germany; 2 Department of Medical Humanities, University Medical Center Utrecht, Po Box 85500, 3508 GA Utrecht, The Netherlands; 3 Department of Psychiatry and Psychotherapy, University Medical Center Göttingen, Von-Siebold-Str. 5, 37075 Göttingen, Germany

**Keywords:** Dementia, Alzheimer’s disease, Advance directives, Research participation, Ethics

## Abstract

**Background:**

Research with persons with dementia is important to better understand the causes of dementia and to develop more effective diagnostics, therapies, and preventive measures. Advance Research Directives (ARDs) have been suggested as a possible solution to include persons with dementia in research in an ethically sound way. Little is known about how people, especially those affected by cognitive impairment, understand and regard the use of ARDs, as empirical studies are mainly conducted with healthy, non-cognitively impaired, participants.

**Methods:**

This qualitative study, a sub-study of a larger study on the evaluation of ARDs in the context of dementia research in Germany, consists of semi-structured in-depth interviews with 24 persons with cognitive impairment.

**Results:**

Our results indicate that most participants consider ARDs a valuable tool for allowing them to make their own decisions. Many would prefer to draft an ARD when they are still healthy or soon after the diagnosis of cognitive impairment. Participants suggested that the completion of ARDs can be advanced with the provision of practical support and increased dissemination of information on ARDs in society.

**Conclusion:**

Persons with subjective or mild cognitive impairment (SCI/MCI) suggested several motivating factors and concerns for completing an ARD. Clinicians need to be trained to accommodate patients’ needs for sufficient and adequate information. Furthermore, a standardised, partly pre-formulated template could be helpful for drafting an ARD. As such tested templates are currently not yet available, this addresses the urgent need for more translational and implementation research for the use of ARDs.

## Background

Doing research with persons with dementia is important to better understand causes of dementia and to develop more effective diagnostics, therapies, and preventive measures. One of the major difficulties of including persons with dementia in research is that due to cognitive decline, at a certain point, they become not (fully) capable of deciding whether they want to participate in research. Up until now, the international legal standard for including persons with dementia who are not able to decide for themselves, is to ask for the consent of a legal representative [1, 2]. This standard is broadly applied, also for other groups of people with cognitive impairment, but has several flaws: representatives often fail to accurately predict the patient’s decision [3] and they can perceive the responsibility of making the right decision as burdensome [4, 5]. Other means of including persons with dementia in research are therefore being explored and the use of advance research directives (ARDs) has become a prominent alternative over the past couple of years.

An ARD is a tool that gives competent individuals the opportunity to express their willingness or objection to participate in clinical research for when they become incapacitated. Therefore, an ARD could allow a person to give direction to their life for a phase when they no longer have the capacity to do so. Proponents of ARDs argue that the moral authority of advance directives is derived from the principle of respect for a person’s (precedent) autonomy [6–9]. The benefit of an ARD is that consent can be based on the preferences and wishes of autonomous persons before they lose capacity to provide informed consent. Furthermore, ARDs could increase the participation rate of cognitively impaired persons or persons with dementia in clinical research by overcoming the reluctance of legal representatives to authorise such participation. At the same time, a serious concern addressed regarding the use of ARDs in dementia research is that persons with dementia have subjective experiences and possibly encounter fears in such research settings, but are not able to express these anymore. The explicit concern is that an ARD may overrule such emotional expressions that do not conform to, or that contradict earlier expressed preferences [10]. How the difference between prior preferences and current wishes is interpreted can have crucial implications for how ARDs are dealt with. Aside from these ethical-philosophical questions, also questions with regard to the implementation have been raised. These affect the uptake of ARDs and their usability for patients.

The NIH allows the use of ARDs in the US, and regulations are in place in Canada and in Switzerland [11]. Although ARDs are legally allowed in these countries, the uptake remains rather low [12, 13]. Currently, ARDs are also attracting increasing scholarly attention within the European context [14, 15]. While the more widely known advance care directives (ACDs) serve the purpose of documenting future health care decisions such as life-sustaining treatments, resuscitation, or organ donation; both ARDs and ACDs are considered documents that record patients’ preferences and decisions [16]. Such directives can also be used to appoint a representative or proxy decision-maker. The role of a legal representative is relevant, as a representative may have to consent also when an ARD is in place and can be regarded as a safety measure for protecting research participants with diminished decisional capacities. Scholars have suggested embedding the drafting of ARDs into the broader process of advance care planning [17]. However, we know very little about how people at risk of developing dementia and early/mild forms of cognitive impairment understand and regard the use of ARDs, as most empirical studies are conducted with healthy individuals [17–22]. The reasons why ARDs are rarely taken up remain unclear, some have claimed it is due to lack of awareness or knowledge [23, 24], or that only few patients are interested in making their own decisions regarding research [25] and prefer to delegate decisions to representatives [26]. There is, however, no empirical evidence supporting these claims. Others have tested motivational interventions for completing ARDs that were only marginally effective [13]. Furthermore, the scarce data available refers to the North American context only and may not apply to Germany, because of cultural and major contextual differences regarding health policy and law. Given the recent changes in German legislation of the Medicinal Products Act (MPA), which requires an ARD to be in place for the participation of incompetent research subjects in research with group benefit, it is ever more pressing to provide guidance on how this law should be operationalised in current clinical practice. The perspectives of those with cognitive impairment are important for operationalising this law in a patient-friendly manner, especially for gaining insight into which factors may contribute to the acceptance and use of ARDs. As the introduced law in Germany demanding an advance directive for research with incompetent research subject was implemented while we were planning the study, we did not ask whether such a law should come into place or whether there should be advance directives at all, but how this law should be operationalised in practice, what kind of help or support people may need and what worries or concerns they may have. In the present work, we investigate how persons with subjective or mild cognitive impairment (SCI, MCI respectively) assess the introduction of ARDs, what arguments they use to support their attitudes, and, in particular, whether they can be motivated to complete such directives.

## Methods

### Data collection

The data analysed in this qualitative interview study is part of a larger study focusing on the introduction of ARDs in Germany and investigates the views of affected people, their caregivers, and health care professionals. Written informed consent was obtained from all subjects. For this sub-study, we included semi-structured interviews of 24 persons with cognitive impairment (see Table 1). As this is the first study that focuses on affected people’s attitudes towards ARDs, we employed an explorative qualitative method. The semi-structured format for the in-depth interviews provided participants with the opportunity to discuss matters they believed needed emphasis, while offering structure throughout the interview (see Supplemental File 1). None of the participants had previously completed an ARD, given that the changes in German law are very recent and due to the fact that ARDs are not yet fully operational in clinical practice and no standardised template exists for the German context to date. The interviewer elucidated the concept of an ARD based on the concept of an ACD as a document that records patients’ preferences and decisions. Given the interviewees’ limited knowledge of ARDs, we prepared an information sheet with elucidations of concepts such as legal representatives, informed consent, advance directives, and forms of medical research in the context of dementia research, which was explained before each interview (see Supplemental File 2). By drawing an analogy with a well-known form of advance directives, an ACD, we intended to clarify the form of an ARD. By outlining alternatives to ARDs, namely proxy decision-making and informed consent, we provided participants with balanced information and had extensive discussions in the research team to ensure neutral language in these forms. Furthermore, we showed interviewees a provisional template of an ARD we created on the basis of existing templates in other countries (North America), to give interviewees an impression of what kind of preferences and options could be documented. The present study was approved by the Research Ethics Board of the University Medical Center Göttingen.

**Table 1:** Participant characteristics

Characteristic	n
Gender	
*Male*	11
*Female*	13
Age (in years)	
< *60*	10
*60–74*	9
*> 74*	5
Educational level	
*High school/practical education*	18
*College*	2
*Postgraduate education*	4
Diagnosis	
*SCI*	13
*MCI*	8
*Not willing to share/not sure*	3

This study was designed to learn about the views of people with cognitive impairment on the introduction of ARDs, as this perspective is currently missing in the literature. None of the authors were involved in the introduced changes in the MPA in Germany. Nevertheless, elucidating the position of the authors with regard to the topic of ARDs is important to clarify their potential influence or interests or lack thereof in this particular study. The first author KJ has previously published more conceptual papers on the use of advance research directives in international peer-reviewed journals. In these papers, she has argued in favour of using advance directives in dementia research from a moral-theoretical point of view. She initiated this empirical research study as she is convinced of the value of empirical research to inform moral-pragmatic arguments and to realise good clinical practice. The second author JP, who conducted the interviews, has a neutral position with regard to advance directives in general including ARDs in the context of dementia. Together with KJ and SiSchi she published a brief report in ‘The Deutsches Ärzteblatt’, which is a weekly German-language medical magazine published in Germany. Here the authors briefly outlined unresolved issues for potential research participants as well as experts in clinical practice regarding the recent changes in German legislation in the MPA [27]. The third author SiSchi, has previously published papers on the ethical aspects of predicting dementia and diagnosing cognitive impairment and implications for later life planning. She has included advance directives in these discussions but takes a neutral stance towards the introduction of ARDs. However, she strongly supports the need for more empirical work in this area. The senior author KR, is a psychiatrist. With the recent changes in legislation, which occurred without much public or academic debate, she recognised the importance of including the perspective of people with cognitive impairment in the operationalisation of this law and therefore strongly supported conducting this study. She has previously written about the clinical challenges of advance directives but has a neutral position with regard to the use of ARDs.

All interviews took place in Germany, either at the interviewee’s home or in a meeting room of the University Medical Center’s department. Using maximum variation sampling [28], participants were recruited by various channels including patient organisations, newspaper advertisement, the university hospital, and snowball sampling to elicit a wide range of experiences with cognitive decline and types of impairments across the disease. Inclusion criteria for the study were: 1) having SCI (mere subjective cognitive impairment, while test results are normal) or MCI (slight but noticeable and measurable decline in cognitive abilities, but no substantial interference in everyday life or independent function). Participants included patients who diagnosed themselves with SCI, and patients who received a diagnosis of SCI or MCI from a treating physician [29], 2) being at least 45 years old when first experiencing cognitive impairment, 3) being competent to provide informed consent. All but three interviews were one-on-one interviews and were conducted in German by JP (MA, PhD-candidate). In three interviews, the interviewee’s partner was present and occasionally participated in the interview when prompted by the interviewee. Persons with severe cognitive impairment or apparent moderate/severe dementia symptoms were excluded from participation in this study as that would have demanded a different research method and approach. In this study, six eligible persons were approached, who were not willing to participate.

The researcher conducting the interviews had prior experience in qualitative studies including interviews. No relationship was established with participants prior to the interviews. The study period lasted until saturation was reached, meaning that no new perspectives or themes were found in consecutive interviews. Interviews examined the following topics: 1) the diagnosis or perceived impairment, 2) dementia research, 3) informed consent and research participation, and 4) the use of ARDs. The interviews were recorded after obtaining consent, transcribed verbatim, and pseudonymised using a code of letters and numbers in the process. Transcribed interviews were not returned to participants for comments and corrections. Verbatim transcriptions of interviews and field notes were compared with audio recordings to check for accuracy.

### Coding and data analysis

The data was analysed by means thematic analysis [30] assisted by the scientific software Atlas.ti™, a tool for the qualitative analysis of large bodies of textual, graphical, audio, and video data. The interview transcripts were peer-coded and revised by three members of the research team. The analysis combined deductive and inductive coding. We started with an a-priori coding scheme to allow for deductive coding based on topics revealed in relevant literature. The interview transcripts were then reviewed by the research team and further emerging codes were added to the coding scheme. Codes and their meanings were discussed among the research team prior to coding for guaranteeing inter-coder reliability (see Supplemental File 3 for the coding tree). The results refer to the following questions of the topic list: How do you assess the introduction of ARDs? Should anyone besides you be involved in drafting an ARD? What is a good time to draft an ARD? What would assist you in drafting an ARD?

The inductive analysis combined methods of close reading and constant comparison; sub-themes relating to attitudes towards the use of ARDs and ways to motivate the interviewees were examined and systematically reviewed for supporting or conflicting evidence concerning emerging themes and codes. The results of this analysis focus on (1) the use of an ARD, (2) helpful conditions and motivations, and (3) worries and uncertainties, which are discussed below. Quotes were translated into English and reviewed for accuracy by two of the authors (KJ & JP).

## Results

### Demographics

For this sub-study, we included interviews of 24 persons with cognitive impairment (see Table 1). Participants were aged between 45 and 85 years, there was an even distribution of gender and 4 participants had attained a higher education degree. Three interviewees were not sure or not willing to share whether they had a diagnosis of SCI or MCI, during the interview it became sufficiently clear that they have a form of cognitive impairment, therefore it was decided to include these interviews in the analysis. For the classification of SCI or MCI in all other interviews, we relied on self-reporting of the participants and this information has not been checked against medical files. Interviews lasted between 16 and 80 min, with an average length of 42 min.

### Attitude towards ARDs

The large majority of the interviewees had a positive attitude towards ARDs and provided a number of reasons supporting their assessment: Several interviewees considered ARDs an instrument to make their own decisions regarding consenting to or vetoing research participation, people without a partner seemed to feel more often positive about the introduction of ARDs. Since the new legal regulation enables participation in non-therapeutic studies tied to the condition of an ARD being in place, several interviewees reflected on the desirability and consequences of participation in such research studies. Some argued that it is important to help others by participating in research or that they value scientific research. Some interviewees went further by arguing that it is *more* important to help others than to benefit from research themselves. A few interviewees stated that they would be willing to help others, as long as research is not burdensome. Others mentioned that they would be more willing to participate in risky research if research were to personally benefit them. However, several interviewees were sceptical that any research would benefit them personally. Therefore, they were rather reluctant to participate in pharmaceutical research and more willing to provide blood samples or additional CT scans advancing the understanding of their cognitive decline’s aetiology. Some participants had negative or ambivalent attitudes towards the use of ARDs, because they did not want to make anticipated decisions, or felt they could not decide for a situation they have not experienced yet (see Table 2).

**Table 2:** Participants' assessment of the value of ARDs

Assessment	Illustrative quote(s)
Positive	I want to decide about my life myself, at least I want to, as long as I can. DBM6
	And again, it is important for me to emphasise, it is more important to help others than myself. DBM8
Negative	I just don’t like to commit myself in advance. DBW7
Not sure/ ambivalent	Partly it is certainly something good, but as we were saying you do not know what will be in a few years, what the situation will be like. DBM3

### Helpful conditions and motivations

Several conditions and circumstances were mentioned that could assist the drafting of an ARD. In the following, we describe our results organised by who should be involved in drafting an ARD, determining an appropriate time, and which preconditions should be in place, according to our interview participants.

#### Who should be involved?

Some interviewees stated that they would be able to draft an ARD on their own. Overall, persons with SCI uttered more often a preference to complete an ARD on their own, when compared to persons with MCI. Nevertheless, a variety of people who could be involved in drafting an ARD were mentioned. Most interviewees desired to discuss their ARD before completing it with a clinician and argued that either their general practitioner, a neurologist, or psychiatrist would be suitable for motivating them to complete an ARD due to an already existing doctor-patient relationship. A few interviewees thought that the researchers themselves would be suitable to ask about ARDs, because of their relevant expertise. Furthermore, several of the interviewees regarded it important to discuss an ARD with their children or partner for a well-conceived ARD with higher interpretability. In this study, more men than women seemed to prefer to involve a family member in deciding about research participation. Some interviewees did not utter specific wishes for who should be involved, but rather argued with preferential character traits and preconditions, such as trust and independence (see also below). A few interviewees argued specifically that employees of one’s health insurance company should *not* be involved due to lack of expertise and perceived untrustworthiness.

#### Timing

Several interviewees argued that the best time to be motivated to draft an ARD is when one is still healthy; as preventative research might still be beneficial at this point and only when one is healthy, one can make up one’s own mind in peace. A few interviewees mentioned that it is best to draft an ARD shortly (between 2 weeks and 2 months) after receiving a diagnosis, because one first needs time to digest the diagnosis and to think about possible participation in research. One person thought ARDs could also be drafted directly at the time of diagnosis with a possible reminder to complete the ARD 2 weeks later. It was also argued by a few that information about ARDs should be available when one is young (er) for example via public campaigns in the media or health magazines.

#### Other preconditions and motivational factors

Several interviewees also addressed other factors influencing their willingness to draft an ARD. Trust was mentioned by many of the interviewees as being essential for drafting an ARD. Trust, it was argued, is based on knowing the professional, by relying on medical expertise, or by ensuring that the professional has no conflicts of interest. Furthermore, it was stressed to have sufficient time to draft an ARD, to discuss the different meanings and formulations with a medical expert, and to be able to ask questions about different types of research. It was also addressed by some that it would be helpful to have a pre-formulated template for an ARD, because most found it difficult to formulate their individual wishes regarding research. In our sample, more women than men seemed to prefer a pre-formulated template. Furthermore, it was argued that a pre-formulated template would prevent miscommunication and might ease physicians’ interpretation of such directives. Further safeguarding was proposed by requiring a representative to monitor research once the ARD is in use (see Table 3).

**Table 3:** Participants’ perceived motivational factors for the completion of an ARD

Motivational factors	Illustrative quote(s)
Discuss with	
No one	When I have to complete such a thing, I can do that by myself. I don’t need anyone else. DBM5
Clinician/ researcher	I’d say the treating physician. DBM2
	They do not have to be physicians but I think that they should have a bit of knowledge, there should be experience. God, that is ... I don’t know. A research institute could actually do this. Why not? DBW6
Family	I would have to talk about it with my partner that is important and I would talk to my children about it. DBW2
Trust	There must already be a trusting relationship so that I can assume he is acting with good intentions. DBW4
Timing/ time	A doctor who has more than two minutes, who listens and explains what he advises, and does not always look at the clock to check how much time has been wasted. DBM7
	There should be some time in between because you have to come to terms with the idea [of the diagnosis]. DBM2
Pre-formulated template	I think it’s better when you have such a template because, I think I would forget so much or will not think of so many things that a template could point out what is important. DBW1
Role representative	My partner should have the last word for when I am suffering from dementia. So that I can hand over responsibility because I can’t bear it anymore. DBM5

### Worries and uncertainties about ARDs

Several interviewees expressed worries and concerns about ARDs. Some addressed reservations due to the anticipatory character of ARDs; the concerns entailed that they might forget what they had agreed upon or that research options will have developed in the meantime – between completion and application of the ARD. Some interviewees worried that they would not be able to withdraw from research once having completed an ARD and argued that withdrawal had to be possible even after consenting with an ARD. Furthermore, some thought that ARDs cannot prevent abuse in research and will not provide sufficient protection. The need for protection was not only mentioned regarding participation but also regarding the use of collected data. A few interviewees also questioned, on an organisational level, whether physicians and other staff will be trained sufficiently to deal with ARDs (See Table 4).

**Table 4:** Participants’ expressed concerns and remaining questions regarding ARDs

Reservations and worries	Illustrative quote(s)
No reservations	No, I don’t think I have any concerns. DBM2
Anticipation	When I have dementia, then I won’t remember what I have decided earlier. DBM5
	In ten years, then it will be outdated again and then it is something completely different again so it is not feasible. So I would not want to commit in advance. DBW7
Withdrawal	Often when something like this starts you can’t stop it anymore, it’s not like the engine of a car. DBW5
Abuse	That the data is not abused, but that one really only uses my data for dementia research. I worry about the pharmaceutical industry. Patients should definitely not be abused by any company, now they can be used as experimental objects. DBW6
Condition for appropriate counselling	The question is whether for this … you will have to train people to sit down and discuss it. DBW12

## Discussion

### Views of persons with cognitive impairment

The current study adds to previous research in several ways. First, we focus on cognitively impaired people’s perspectives on the use of ARDs, whereas other studies have focused on healthy adults. Persons with mild cognitive impairment have an increased risk of developing dementia [31]. Most persons with dementia, at an earlier stage of their life, were able to provide consent to research participation. The often slowly progressive nature of dementia raises the possibility for patients to anticipate their inability to provide consent in the future. ARDs therefore are considered a potentially useful tool, especially for this population. Second, we explore which factors can motivate people to draft an ARD. In this sample of 24 cognitively impaired people, most assessed ARDs positively as a tool to maintain their self-determination and to alleviate the burden when decisions have to be made by representatives. This finding differs from earlier findings of studies conducted in the US and the Canadian context that indicate older adults’ willingness to give leeway to their proxies to overrule previously made decisions [18, 20, 32]. While most participants were not aware of the details of the legal changes in Germany, our interview study raised their interest and involvement in the topic, hence engaging them in the discourse and possibly contributing to their positive assessment of such directives. Particularly interesting within this sample is that several participants stressed that they consider it more important to help others than to benefit from research themselves, as non-therapeutic intervention research was previously prohibited in Germany. It is only recent and since the introduction of the new law that participation in such trials is allowed if an ARD is in place. Furthermore, people without a partner felt more positive about the introduction of ARDs. This is relevant, as the number of older adults living alone is continuously increasing [33]. People living in partnerships may assume their partner will automatically take responsibility for decisions when they are no longer able to do so. Interviewees with higher levels of education tended to have fewer reservations about ARDs but preferred counselling to discuss implications with a trained clinician. Furthermore, several motivational factors were stated such as: being trusted to fill out the directive by oneself, support or counselling of a trusted and informed physician, being asked shortly after receiving the diagnosis, and provision of pre-formulated templates, which seems largely in line with motivating factors for other types of advance directives [34].

Some interviewees mentioned concerns that can be addressed by implementing additional safeguards when conducting research with this population. Such safeguards include recruitment within a trusted patient-physician relationship and offering adequate protection and additional governance for guarding participants’ privacy. One remaining uncertainty concerns withdrawal from research. This indicates the urgent need for legislators and researchers to provide additional safeguards aside from ARDs. One option could be to involve legal representatives to monitor participants who lack decisional capacity for signs of undue burden or objection, but it needs to be investigated how such safeguards can also be applied for people without a partner.

### Clinical implications and recommendations from this study

This study indicates several factors that can motivate persons with cognitive impairment to complete an ARD. Our study indicates that people’s preferences, assessments, and remaining uncertainties vary regarding ARDs. Therefore we strongly recommend to employ a personalised approach to ARDs. Furthermore, our results suggest several recommendations for the consent procedure of research with research subjects who lack decisional capacity and for the implementation process of ARDs. ARDs are considered acceptable tools for consenting to research but require expertise of treating physicians concerning dementia-research methods such as prospective scenarios to support patients and research participants; and clinicians need to be trained to accommodate patients’ and research participants’ fears and needs for sufficient and adequate information. Talking about ARDs, entails talking about the anticipated course of the disease including losing the ability to provide consent. As mentioned by many of our interviewees, physicians should take time to talk to their patients/research participants and their relatives/caretakers – oftentimes overwhelmed by the diagnosis – about such sensitive issues. This poses a challenge for the daily routine of hospitals and outpatient clinics necessitating practical adaptation. As interviewees stated, a standardised, partly pre-formulated template would be helpful for drafting an ARD. As such tested templates are currently not yet available, this addresses the urgent need for more translational and implementation research. As standardisation risks that the document’s individual character is lost, templates should be developed to allow for individualised wishes and adaptations.

### Limitations

Our results must be interpreted in the context of the following limitations. This is a qualitative study, which aims at exploring the manifold reasons and concerns of affected people. Qualitative methods are always subject to bias, in the sense that these methodologies are interpretive and exploratory in which the researchers themselves are the instrument to collect and interpret the data. In order to make this process and systematic analysis as transparent as possible, the topic list, coding tree and information sheet are available as supplemental files. The systematic analysis has been enabled by continuous critical reflection within the research team, who are all trained in qualitative research methods. Given that research on this topic is very rare to date, this study aimed at providing first directions that need to be further explored in subsequent studies. Thus, our study can be understood as a step towards the development of an implementation strategy and also as a starting point for studies testing whether the identified factors in this study may indeed be effective motivators for the uptake of ARDs. Longitudinal studies are necessary to further explore affected people’s attitudes towards ARDs, analysing whether preferences may change over time.

We provided the interviewees with an information sheet and a provisional template in order to facilitate a conversation about topics they were not (very) familiar with. This may have affected the ways in which our participants perceived and evaluated the use of ARDs. Nevertheless, given that most interviewees discussed both the positive aspects of ARDs as well as concerns, we believe that we managed to provide balanced information, thereby reducing information bias. Furthermore, our interviewees were self-selected and possibly more than average open to discuss dementia research participation. This may limit the applicability of these results to populations with different backgrounds. Also, the participants were fairly low educated, this stands in contrast to other studies often over-representing higher educated people. Further, our sample can be considered rather homogeneous with regard to the interviewees having a German cultural background. We did not enquire about ethnicity or migration background in our study, as this is not common in German studies. Nevertheless, we believe that a cross-cultural analysis would be interesting for future studies with larger samples.

## Conclusion

This study with cognitively impaired participants shows that they assess ARDs mostly positively as a tool to maintain their self-determination and to alleviate the burden when decisions have to be made by representatives. Several interviewees uttered concerns about the possibility to withdraw from a study after signing an ARD. Therefore, interviewees made clear that additional safeguards have to be in place for them to be willing to participate in dementia research. Furthermore, this study indicates that persons with cognitive impairment can be motivated to complete an ARD, when a suitable person is involved, shortly after the diagnosis, in a situation with sufficient information and trust. This means that the drafting and completion of ARDs can possibly be increased when practical support is provided and information about ARDs including pre-formulated templates are widely spread. There is an urgent need for studies testing such motivational approaches, also taking cultural differences into account.

Researchers and clinicians will have to adopt ARDs in their clinical practice, as these will be increasingly implemented in Europe. The implementation of ARDs in Germany increases the necessity to further explore affected people’s assessment of this tool in advance prior to implementation and to develop frameworks for successful introduction into clinical practice.

## Supplementary information

**Additional file 1.** Interview guideline for individual interviews on the topic of ARDs.

**Additional file 2.** Information sheet.

**Additional file 3.** Coding tree.

## Data Availability

The data that support the findings of this study are available on request from the corresponding author KJ. The data are not publicly available due to them containing information that could compromise research participant privacy.

## Abbreviations

ACD: Advance Care Directive
ARD: Advance Research Directive
MCI: Mild Cognitive Impairment
MPA: Medicinal Products Act
SCI: Subjective Cognitive Impairment
