# Supplementary material for: Motivations for people with cognitive impairment to complete an advance research directive – a qualitative interview study
Source: BMC Psychiatry. 2020 Jul 8;20:360. doi: 10.1186/s12888-020-02741-7 (PMC7346429; doi:10.1186/s12888-020-02741-7)
Supplement: Supplementary file 1 — Additional file 1. Interview guideline for individual interviews on the topic of ARDs. [file 12888_2020_2741_MOESM1_ESM.docx]

| Universitätsmedizin Göttingen, 37073 Göttingen |  | Zentrum Psychosoziale Medizin  Institut für Ethik und Geschichte der Medizin |
| --- | --- | --- |
| Abteilung Ethik und Geschichte der Medizin, Prof. Dr. Silke Schicktanz, Humboldtallee 36, 37073 Göttingen |  |  |
|  |  |  |
|  |  |  |
|  |  | **Projektmitarbeiterin:** |
|  |  | **Adresse**  **Telefon**  **Fax**  **E-Mail** |
|  |  | Date |

**Interview guideline for individual interviews on the topic of ARDs**

**DIAGNOSIS**

1. Could you tell me why you first made an appointment at the memory clinic and how you experienced your visit there?
2. Has your visit to the memory clinic (or the received diagnosis) had any influence on your personal or social life?
3. Have you considered possibilities to plan your future in light of your diagnosis? (have you discussed this with your family, have you heard about a so called ‘power of attorney’, do you know about advance directives or proxy directives?)

**RESEARCH PARTICIPATION**

**INFORMATION FILE: Explain the differences between research & care, types of research (observational studies & intervention studies, longitudinal & short-term studies.**

1. Have you, aside from this interview study, ever participated in a research study?
   If yes: What was your main motivation to participate, how did you experience your participation?
2. What do you think about people with dementia or people with cognitive impairment participating in (bio)medical research? (is it acceptable/if so under which conditions?)

**INFORMED CONSENT**

Informed consent is an important requirement for the participation in scientific research (as you have also just experienced for yourself - with me explaining the aims of this study and with you filling out all of these forms). In order to provide informed consent, you have to be able to decide for yourself, determine whether you are willing to participate and be able to communicate your choice.

People in more advanced stages of dementia are often not able to provide such informed consent anymore. In this scenario there are two options for consenting to research participation:

1. A proxy or legal representative decides on behalf of the affected person at that given time

OR

1. One anticipates willingness to participate in future studies and puts the decision into writing (in the form of an advance directive)
2. Which option do you prefer, and why?

**ARDs**

Until now, proxies or representatives have made decisions on behalf of the person with cognitive impairment, if the person was not able to decide for him/herself anymore. Recently, advance research directives have been introduced by the government that have similarities to advance care directives. These ARDs mean that one could write down anticipated decisions about whether one is willing to participate in dementia research, for a phase when one is in a more advanced stage of dementia and is not able to communicate one’s own wishes anymore.

1. What do you think of the option of drawing up an advance directive for the research context?
2. If you were to draft such a directive yourself, what would you want it to entail? Which information would you need to anticipate such a decision? (What would you be able to anticipate? What is most important for you? 🡪 show example of ARD template)
3. Who should ask you, whether you are interested in drafting an ARD? (GP, clinician, researcher, family, nurse, health insurance…). What would be a suitable moment in time for such a question?
4. Risk/burden: participating in research always entails a certain burden for the participant and sometimes there are also risks involved – think, for example, of extra medical examinations, taking blood samples or, for example, a time investment (such as in interview studies). In intervention studies with experimental drugs, side effects may also occur, e.g. nausea, kidney failure or allergic reactions. What would be acceptable for you, when you think of research participation? (And do you think your preference (choice) could change, e.g. if you were no longer able to provide consent?)
5. Individual vs. Group benefit: When deliberating about filling out an ARD for the participation in future medical research, does it matter for your decision whether you benefit from participation personally or whether your participation may only potentially benefit others?

[e.g.: Observational studies on behavior (e.g. how care affects well-being or certain patterns of activity or taking experimental drugs and tablets to be tested, which potentially could benefit you)

1. Do you see benefits in drafting an ARD, (or) do you have concerns?

Would you regard the drawing up an ARD as a form of easing a burden?

a. If you have concerns: which ones? Please specify your concerns.

b. What would help you decide on whether or not to prepare an ARD for yourself? What kind of help/support would you want and need specifically?

1. In your opinion, what should be the role of a proxy or a representative, in general and regarding research participation? What should the responsibilities of this person entail? Would you allow this person to overrule your anticipated decision?
