# Supplementary material for: Motivations for people with cognitive impairment to complete an advance research directive – a qualitative interview study
Source: BMC Psychiatry. 2020 Jul 8;20:360. doi: 10.1186/s12888-020-02741-7 (PMC7346429; doi:10.1186/s12888-020-02741-7)
Supplement: Supplementary file 2 — Additional file 2. Information sheet. [file 12888_2020_2741_MOESM2_ESM.docx]

**INFORMATION SHEET**

| **Representative**  If you are not able to make your own decisions anymore, a person, who you know well, can function as a representative to make decisions on your behalf. The task of a representative is to make decisions that are in line with your own will and wishes. | |  |
| --- | --- | --- |
| **Informed Consent**  Informed consent is important for participation in research studies. The participant is informed about the study in order to make an informed decision about his/her willingness to participate. Only people who can voluntarily and competently decide for themselves and can understand the provided information, are allowed to consent to research participation. | |  |
| **Advance directive** An advance directive is a document in which anticipated wishes and one’s will can be described and put down in writing. This document can record your will, also for a time when you are not able to express it yourself anymore. Such a document entails information on medical interventions, such as medical treatment. | |  |
| **Medical Research**  Phycisians often conduct medical research in order to gain new scientific insights. In research, the primary goal is to collect data, for example, via observation or interventions (such as by taking blood samples or by administering experimental treatments). Participation in research involves a burden for you. Risks and benefits are still being studied and are often unknown before the study begins. There are studies that can directly benefit you and there are studies that only have the potential to benefit other people in the future. | |  |
| **Examples of research studies** |  | |
| **Observational studies** | **The researcher observes your living conditions or those of your relatives, e.g. the living situation, the course of symptoms over a certain period of time or the influence of music therapy. You yourself have no direct benefit from such studies. No risks are involved.** | |
| **Intervention studies** | **Intervention studies actively change something in your current situation, e.g. by prescribing medication and by taking (several) blood samples, or by performing brain scans or memory tests. This allows to examine the effectiveness of various treatment options, such as the effectiveness of medication. These studies may have a benefit for you. These studies always entail risks.** | |
| **Longitudinal studies** | Groups of people are studied for a certain period of time. The study is carried out by measuring the same people at different points in time, to compare potential changes over time. | |
| **Short studies** | Short-term participation, one-time event. | |
