# Supplementary material for: Motivations for people with cognitive impairment to complete an advance research directive – a qualitative interview study
Source: BMC Psychiatry. 2020 Jul 8;20:360. doi: 10.1186/s12888-020-02741-7 (PMC7346429; doi:10.1186/s12888-020-02741-7)
Supplement: Supplementary file 3 — Additional file 3. Coding tree. [file 12888_2020_2741_MOESM3_ESM.docx]

Supplemental file 1: coding tree
